# Kinematic determinants of scoring success in the fencing flick: Logistic and linear multiple regression analysis

**DOI:** 10.1371/journal.pone.0222075

**Published:** 2019-09-25

**Authors:** Anya N. Michaelsen, Corey L. Cleland

**Affiliations:** Department of Biology, James Madison University, Harrisonburg, VA, United States of America; Toronto Rehabilitation Institute - UHN, CANADA

## Abstract

Sport fencing is an open-skilled combat sport practiced around the world. Although previous research addressed kinematics of the lunge and fleche, there are currently no studies on the flick. The flick is a high-level action that involves bending the blade toward the opponent, much like a whip or fly-fishing cast. The aim of our research was to identify the kinematic variables that significantly influence scoring success in two elite foil fencers. In particular, we asked what aspect of the movement each *individual* fencer can *change* to improve their likelihood of scoring. Two elite foil fencers of similar skill were instructed to execute flicks at a dummy target that mimicked the opponent’s shoulder. High speed video (650 fps) captured the motion of the tip of the foil, blade of the foil, and limb joints; the latter were used to calculate joint angular velocities, hand height and distance throughout the flick. Scoring success was determined with a conventional scoring box. Our results showed that the two fencers exhibited significantly different kinematics, coordination and scoring. Using three complementary regression approaches, we showed that each fencer could improve scoring by changing specific aspects of their kinematics. For fencer A, only improvement in consistency in distance from the target would improve scoring. For fencer B, the changes were more complex. In addition to improvement in consistency in distance, fencer B could also increase (finger, wrist) or decrease (shoulder) joint angular velocity or improve consistency of limb joint angular velocities. Unexpectedly, and in contrast to common coaching practice, hand height had only a weak effect, possibly because both fencers had learnt to keep their hand high at the end of the action. In summary, our results emphasize that coaching of elite fencers should be individualized.

## Introduction

Modern sport fencing is an open-skilled combat sport [1,2] practiced around the world whose aim is to score touches by hitting the opponents’ target area with the weapon. There are three different weapons–foil, epee and sabre–which differ in blade design, scoring target, and tactics. Much of the research conducted on fencing (reviewed in [1,3–5]) has focused on the medical (e.g. [6–10]), anthropometrical and physiological (e.g. [9,11–14]), and gender (e.g. [10,15,16]) aspects of the sport; fewer papers have addressed the limb and body kinematics during specific fencing actions (commented on in [1,17]), which are under the fencer’s direct control and reflect both tactics and neuromuscular coordination [17–20]. Further, most previous research studied novice fencers [11,21], compared elite fencers to novice or non-fencers [19,20,22,23] or the characteristics of elite fencers [18,24–29]; few have studied the differences between elite fencers [25]

**Fig 1:**
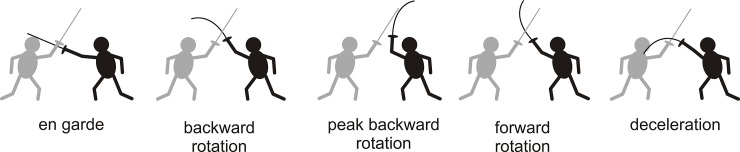
Phases of the fencing flick. Phases of the flick, delivered by the fencer on the right (black filled) against the fencer on the left (gray filled), are illustrated by a cartoon. En garde: The initial posture prior to commencing the flick. Backward rotation: The base of the blade is rotated backward, causing the blade to bend forward. Peak backward rotation: The backward rotation of the base of the blade stops causing the blade to bend backwards. Forward rotation: Rapid forward rotation of the base of blade causes the blade to further bend backwards. Deceleration: The forward rotation of the blade is abruptly stopped, causing the blade to markedly bend forward toward the target. If contact is made with adequate force a point, or touch, is scored.

Kinematic studies of the differences between elite foil, epee and sabre fencers have largely focused on the lunge [17,30] and, to a far lesser extent, the fleche [25]. In contrast, and in spite of its importance to modern fencing [4], there have been no experimental studies of the flick. The flick is a high level foil action [31,32] that involves rotating the blade backwards first, then rapidly rotating toward the target (Fig 1). During its descent, the base of the blade is then abruptly decelerated causing the tip to bend towards the target, much like a whip [33] or fly fishing cast [34]. When the tip hits the target, the blade exhibits a pronounced arc [31,32]. The flick is a difficult action because it requires upper body strength, coordination, and accuracy, and thus is usually only observed at high levels of skill. Reasons for learning and using the flick are that it allows a fencer to hit their opponent *around* the opponent’s parry or block, and that it opens up new targets, such as the shoulder, back, and sides, which are almost always inaccessible with a simple lunge [32].

In summary, there is a complete absence of research on the fencing flick and limited or a lack of studies incorporating blade kinematics and scoring [12], high-speed video greater than 600 fps, comparison between elite fencers using different tactics, and computer simulations. Consequently, the aim of our research was to determine the kinematic variables of the elite fencers’ limb that impacted scoring success in the flick; in other words, what aspect of the movement can the individual fencer *change* to improve the likelihood of scoring? Since coaching methodology champions hand height, our primary hypothesis was that the final height of the hand would significantly increase the scoring success of the flick.

## Materials and methods

Three methods were used to determine the significant limb kinematic variables. The limb directly impacts the movement of the blade, which directly controls the movement of the tip, whose interaction with the target determines success. Thus, the first approach to identifying the relevant limb variables was to use multiple linear regression to work backward from the tip to the blade and finally to the body. The second approach was to use multiple logistic regression to directly identify limb variables that influence scoring success. Finally, a computer simulation using forward dynamics and multiple linear regression was used to evaluate the effect of angular limb variables on a proxy for scoring success, tip velocity.

### Participants

Participants were two highly rated fencers with “B” or “C” ratings (top ~4% of competitive fencers in the United States; ratings range from a high of A through B, C, D, E and unrated; fencer B was nationally ranked at 21st in women’s foil age 14–15 in the United States, fencer A was unranked at the time of the study). Both participants were intentionally similar; female, mid-teens, had at least five years of fencing experience, used medium stiffness blades and had prior experience flicking in practice and competition. Although their weights (fencer A 59 kg, fencer B 54 kg) and heights (fencer A 175 cm, fencer B 168 cm) were somewhat different, their body bass indices (19.3 and 19.1 kg/m2 respectively) were similar. Written informed consent was obtained from each participant. The study was approved by the Institutional Review Board at James Madison University (14–0158). Experiments were conducted at a local fencing club, which agreed in writing to the use of its facilities and equipment.

### Fencing task

In foil fencing, scoring success is dependent on two conditions being met: the tip of the foil must be both depressed and simultaneously in contact with the opponent’s target area defined by a metallic lamé vest. To adequately compress the tip, a force of at least 4.9N (500g) is required for a duration of at least 15ms. If both of these conditions are met, a conventional scoring box will display a colored light to indicate success. If the tip is depressed but not in contact with the target, the box will display a white light. If the tip is not depressed, no lights will be displayed. For this study, a colored light (i.e., both criteria were met), was categorized as a “hit”, and a white light or no light (i.e. only one or none of the criteria were met; occurred infrequently) was considered a “miss”.

**Fig 2:**
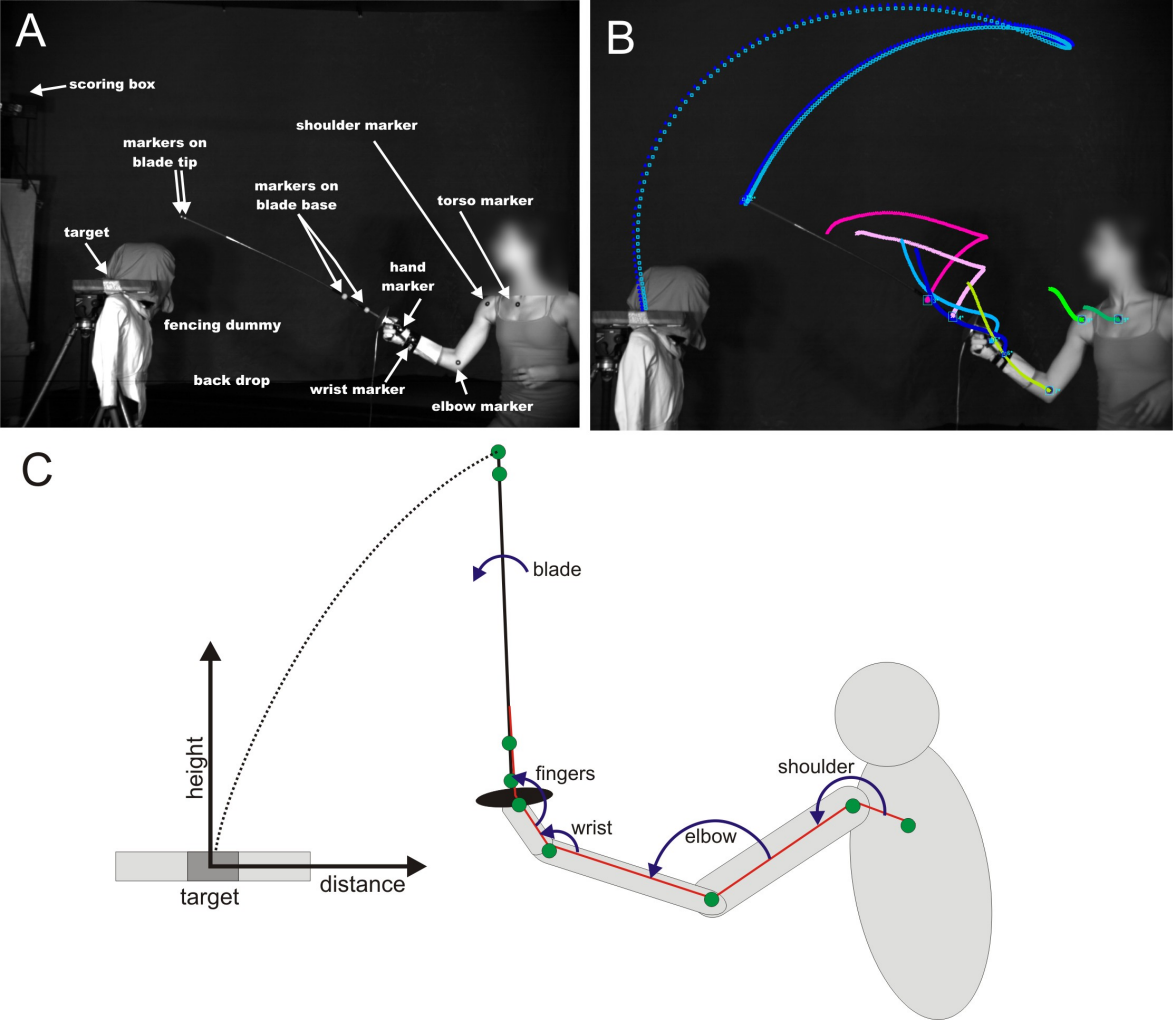
Methods. (A) Single video frame from the en garde phase of the flick shows the experimental arrangement of the nine tracking markers, scoring box, fencing dummy, back drop and target. The 5 cm target was created by electrically conductive lamé material wrapped around a 30.5 cm long aluminum plate covered in non-conductive fabric. (B) Single video frame with superimposed tracked trajectories of the 9 markers over the entire duration of flick. (C) Diagram illustrates the Cartesian coordination system whose origin is at the center of the target and the calculated joint and blade angles. Green circles are markers and red lines highlight the limb-blade kinematic chain. The direction of joint angle arcs indicate increasing angle.

Since real bouts have a high amount of variability and added unknown factors, our study was performed in a controlled environment with two-dimensional motion, a dummy opponent and an artificial target. Participants were instructed to adopt an en garde position at a comfortable distance from a dummy that was dressed in fencing gear to simulate a real opponent (Fig 2A).

### Target

In foil fencing, the target area covers the entire torso [2], demarcated by a metallic vest, or lamé, worn by the fencer. Targets for the flick include the leading shoulder, back, and flank. In order to reduce variability, we restricted the target to the top of the right shoulder, a common target for the flick in foil fencing. However, even within the confines of the shoulder, variability manifests itself through differences in slope of the back, top and front portions of the shoulder, as well as the stiffness, and height. To diminish these variables, we created a substitute target approximately the same size, height, and stiffness of the shoulder.

Since the target was an approximation to the top of the shoulder, it was placed just over the dummy’s shoulder (Fig 2A). Although the substrate on which the target was placed was 30.5cm in length and extended in front and behind the shoulder, only the center portion that approximated the shoulder was covered with electrical conductive lamé fabric to record successful hits. The area of the metallic target thus approximated the portion of the shoulder that would be roughly planar, which was found to average 6.4 x 10.2 cm across the two fencers.

**Fig 3:**
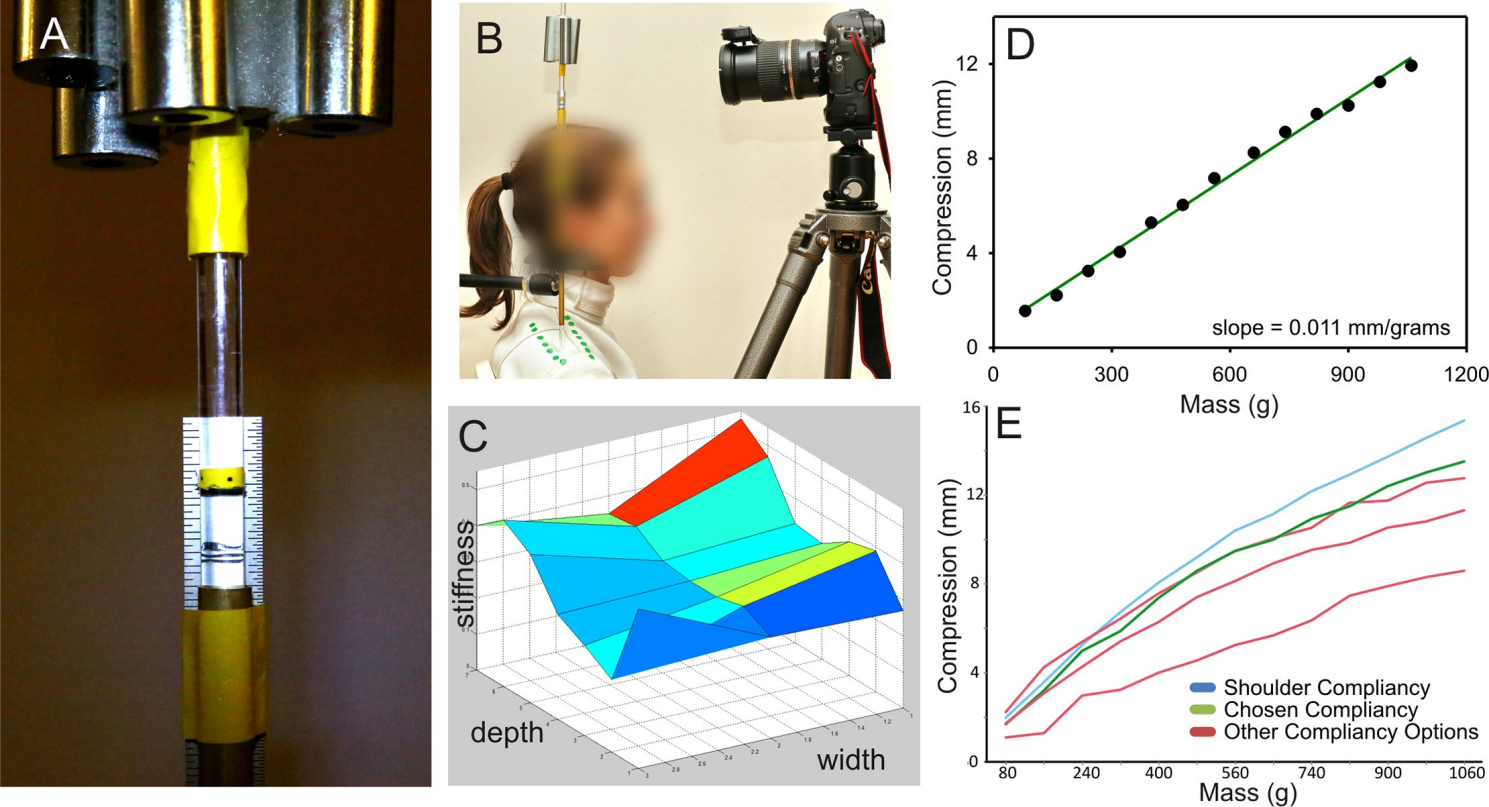
Stiffness measurement and target construction. (A) Lab-made device to measure stiffness. Weights are at the top and height is measured by the location of the black dot relative to the ruler. (B) Arrangement for applying the weighted cylinder to the shoulder and recording by camera. (C) Stiffness across the shoulder was relatively constant. (D) Average compression and mass over all locations. Stiffness across the shoulder was relatively constant. (E) Comparison of the average stiffness for the shoulder (blue), several candidate rubber surfaces (green and red), and the one chosen (green).

Given the importance of the tip-target interaction due to the need to adequately depress the tip, approximating the stiffness of the shoulder was crucial; a softer material could create lower peak forces, and a firmer material could create higher peak forces. In order to measure stiffness, we created an apparatus that allowed weights that created forces similar to those that occur during fencing (>4.9 N, 500 g) to be applied incrementally to a vertical acrylic rod (Fig 3A) and the resulting compression was determined by photographing an attached ruler (Fig 3B). When compression was graphed against applied mass, the slope of the line was the stiffness. Using this device, we measured stiffness in 21 equally spaced locations (3x7 array) across the top of the shoulder (Fig 3C). The results were then averaged to provide the overall stiffness of the shoulder (Fig 3D; 0.011 mm/g).

To find a material to emulate the shoulder’s elastic qualities, we created several single and double combinations of 1.27 cm foam (1–50 PSI at 25% defection, “soft” polyurethane; McMaster-Carr). We then measured the stiffness of these foam combinations using the device and compared the results to the stiffness of the shoulder. The foam combination whose stiffness was closest to that of the shoulder (two layers of 1.27 cm, 1–5 PSI “soft” polyurethane; 0.095 mm/g) was selected to be used in the target (Fig 3E). The foam was then covered in light cloth and a portion of it, described above, was covered in metallic lamé material to define the scoring portion of the target.

### High speed video recording

The camera (650 fps, 2336x1728 pixels, 5 GB memory, 25 mm lens, F /4.0, shutter speed 1/8000 s, NR5S1, IDT, Pasadena, CA,) was placed orthogonally to the plane of motion of the blade, and levelled such that gravity was vertical using a plumb line. High frame rate was needed to capture the rapid movement of tip of blade and the fast shutter speed minimized blurring. High speed video was recorded while the fencer was connected to a standard scoring box used to register touches. The camera was set to post-trigger, recording the three seconds preceding the touch. Immediately after a fencer performed a flick, the camera was manually triggered through a computer interface, which allowed the fencers to perform the flick naturally when they felt ready. Video data was recorded directly to a Personal Computer laptop hard drive.

To provide a uniform and high contrast background for video tracking, a 914 x 488 cm black velour backdrop was hung behind the fencer and target/dummy. Furthermore, to enable the use of such a short shutter speeds, high intensity lighting was provided by two conventional shop halogen lights at 1000 W each placed about 4 meters lateral to the fencer.

### Procedure

In order to track the movement of the tip of the blade, base of the blade and limb, nine markers were placed on the fencer’s blade and body (Fig 2A). Two markers (4mm in length) were placed on the tip using small stripes of reflective white tape. Two small white Styrofoam balls (1.27 cm diameter) were threaded over the blade near the base of the blade and held in position by friction. High contrast black/white markers were placed on the hand, wrist, elbow, shoulder and chest to mark the limb and torso.

The target was placed over the shoulder of a dummy, which was dressed in full fencing attire to simulate an opponent. The fencers warmed up normally and were allowed practice time to adjust to the modified target. Fencers used their own blade and glove to provide a more natural environment for them. Both fencers used a pistol grip.

The fencers were given the verbal cue “en garde, ready, fence”, wording commonly used by referees in competition to signal the beginning of an engagement. After the cue was given, the fencer could perform the flick once they felt ready, and the video was recorded using a post-trigger. In order to insure a similar and sufficient number of both successful (“hit”) and unsuccessful (“miss”) trials for statistical analysis, for each participant similar numbers of successful and unsuccessful trials were collected by alternating the desired result for each trial, such that following the recording of a “hit”, the next trial would have to be a “miss”. All attempts were recorded, however only attempts that had the desired, alternating, result were saved for kinematic analysis. The fencers were provided at least one minute between trials, regardless of whether or not they were saved. Sixty-seven trials were collected for participant A (34 hits, 33 misses) and seventy-eight trials were collected for participant B (39 hits, 39 misses).

### Analysis

Following the experiment, each marker was automatically tracked throughout the flick in ProAnalyst (Fig 2B; Xcitex, Woburn, MA). Data were then imported into the programming environment Matlab (Mathworks, Natick, MA) for the calculation of kinematic trajectories using custom routines written by the authors (Fig 2C).

Variables that might be expected to influence the success of the flick, especially including those typically identified by coaches, were calculated (Table 1). Regarding tip variables, based on the scoring requirement that the foil tip contact the conductive lamé target with adequate force (>4.9 N, 500 g), the *tip distance*, *tip velocity* and *tip angle* at contact were determined based on the most distal tip marker. Since the last frame prior to contact was, even at 650 fps, above the target, the tip variables were calculated based on extrapolation to tip contact. “Stepping” back from the tip, the blade variables that could influence the tip variables were *blade distance* at tip contact, peak *blade angular velocity*, and peak *blade height*. Stepping back further to the limb variables, six were considered, all of which are under direct control of the fencer. Peak s*houlder angular velocity*, peak *elbow angular velocity*, peak *wrist angular velocity* and peak *finger angular velocity* would be the primary determinants of both angular velocity and distance of the blade. The “finger” angular rotation, although not around a physical limb joint, arose from the loose coupling between the hand and the base of the blade, allowing the fencer to change the angle of the blade without changing the angle of the hand by individual finger movements. The last two limb variables–peak *hand height* and *hand distance* at tip contact–also determine blade kinematics. Hand distance was taken at the wrist marker to avoid variability resulting from hand rotation.

**Table 1:** Variables.

	Variables	Reflected variables	Fencer A	Fencer B
scoring	scoring (0/1)		58%	44%*
tip	velocity	Reflected velocity (cm/s)	2.92±0.45 (1.59–3.90)	2.22±0.54 (1.14–3.41)*
	distance	Reflected distance (cm)	0.19±1.78 (-3.24–5.35)	0.10±2.00 (-3.2–6.14)
	angle	Reflected angle (deg)	35.7±3.66 (22.7–44.4)	32.3±4.30 (23.3–46.5)*
blade	angular velocity	Reflected angular velocity (deg/s)	1.42±0.10 (1.19–1.63)	1.47±0.12 (1.24–1.77)
	height	Reflected height (cm)	22.1±1.85 (17.3–26.4)	19.3±2.92 (11.4–27.5)*&
	distance	Reflected distance (cm)	66.3±1.1 (64.3–69.4)	75.3±2.14 (71.7–81.8)*&
limb	finger angular velocity	Reflected finger angular velocity (deg/s)	0.31±0.10 (0.10–0.54)	0.50±0.14 (0.17–0.85)*
	wrist angular velocity	Reflected wrist angular velocity (deg/s)	0.97±0.06 (0.87–1.12)	1.33±0.13 (0.99–1.56)*&
	elbow angular velocity	Reflected elbow angular velocity (deg/s)	5.17±0.49 (4.28–6.65)	3.70±0.44 (2.92–5.88)*
	shoulder angular velocity	Reflected shoulder angular velocity (deg/s)	-2.42±0.21 (-1.90–3.03)	-2.15±0.27 (-1.57–2.98)*
	height	Reflected height (cm)	18.9±1.83 (14.3–23.2)	13.6±3.27 (5.88–23.2)*&
	distance	Reflected distance (cm)	80.8±1.27 (76.0–83.7)	95.1±0.54 (1.59–3.90)*&

Table 1 lists the scoring, tip, blade and limb variables that were evaluated for their impact on kinematics and scoring. Continuous variables were evaluated based on both their original and reflected form. Scoring is represented by 1 (scoring successful) and 0 (scoring not successful). Statistics are mean, standard deviation and range for each fencer for non-reflected variables and percent scoring success for scoring. Asterisks represent statistically significant differences (13 t-tests with Bonferroni corrections for alpha and Chi^2^ for scoring) between fencers for non-reflected variables. Ampersands represent statistically significant differences (13 t-tests with Bonferroni corrections for alpha) between fencers for reflected variables.

There are two ways that any variable could alter a dependent variable. For illustration, the final tip distance at contact with the target could influence scoring success in in two ways. First, increases in tip distance could result in altered (increased or decreased) scoring probability. Regression of tip distance versus scoring would test this hypothesis. Second, consistency, or deviations from the mean for final tip distance in either direction (closer to or further from the target), could result in altered scoring. In other words, both overshooting and undershooting the target would lead to a miss. To test this possibility, the final tip distance was reflected around the mean distance (by subtracting the mean and taking absolute value) of successful trials prior to regression. Thus, all of the tip, blade and limb variables were tested in both their original and reflected state.

### Statistical analysis

We grouped the variables into three sections: tip, blade, and limb. For the indirect approach, we used logistic and linear regression to identify which variables of the tip *statistically* (based p-values) and *functionally* (based on effect sizes) influenced scoring success; second we identified the variables of the blade that influenced the variables of the tip that were found in the first step; and thirdly we identified the variables of the limb that influenced the significant variables of the blade. For the direct approach, we used logistic regression to examine the variables of the body that *directly* influenced success. For the third, computer simulation approach, only angular joint rotation variables (shoulder, elbow, wrist and finger) were considered; the procedure is described fully below.

Angular velocities were calculated as differences between adjacent frames and smoothed by low pass filtering (10 Hz, Matlab “lowpass” function, 0.85 steepness). Variables that were not normally distributed (Shapiro-Wilk, p>0.05), which were all the reflected variables but none of the un-reflected variables, were transformed by a square root operation to achieve verified linearity. Because variables differed in their units and in order to compare slope coefficients, variables were conventionally (subtract mean, divide by standard deviation) standardized. Lack of multi-collinearity was verified by the variance inflation factor (<4.0 for all variables). Following regression, homoscedasticity was verified with a constant variance test and visually based on a scatterplot of residuals.

Multiple regressions, logistic for dichotomous variables (scoring success) and linear for continuous variables (remaining variables), were conducted in a forward step-wise manner to remove statistically insignificant variables. Variables were eliminated in step-wise regression based on F (linear, F-to-enter 4, F-to-remove 3.9) and p values (logistic, p>0.05). In addition, significant variables were removed if their standardized slope coefficient (measure of effect size) was less than half the maximum for that multiple regression (this only occurred once, for the effect of reflected angular wrist velocity on angular blade velocity). All non-reflected variables were regressed together, then all reflected together; finally only the best (lowest p-value) of each non-reflected/reflected pair was retained to avoid multicollinearity.

The coefficient of determination for multiple logistic regressions was quantified by a pseudo r^2^ (Nagelkerke). The coefficient of determination for multiple linear regression was quantified by an adjusted r^2^. The magnitude of effect of variables in multiple logistic regression was quantified by their odds ratio (or inverse odds ratio). Odds ratios were inverted (inverting to convert less than to greater than 1.0) in some instances to facilitate magnitude comparison. The magnitude of effect of variables in multiple linear regression was quantified by their standardized slope.

Tracked and calculated data were stored and analyzed in Excel (Microsoft) and Matlab files. Statistics were calculated in Sigmplot (Systat, San Jose, CA, USA), SPSS (IBM, Armonk, NY, USA) and Matlab. Graphics were created in Sigmaplot, SPSS, CorelDraw (Corel, Ottawa, CA) and Matlab. Small p-values were truncated at 0.0001. Statistical significance was set to p = 0.05. No corrections were made for multiple comparisons except as noted.

### Computer simulation

Although multiple regression computationally separates out the effects of shoulder, elbow, wrist and finger angular velocities, any non-linear interactions will create variability. Consequently, we developed a novel computational simulation in which three of the angular limb variables were held constant while allowing the remaining 4^th^ variable to change. The simulation then calculated final tip velocity as a proxy for scoring success for each trial. Finally, regression was used to evaluate the relationship between each of the eight (finger, wrist, elbow, shoulder, and their reflected versions) isolated variables and tip velocity.

The foil was modeled in Matlab/Simulink/Multibody discretely as 10 segments connected by revolute joints incorporating rotational stiffness and damping. The mass, stiffness and damping were set to values that resulted in foil rotation that approximately matched recorded foil movements. For three of the four limb angular variables–shoulder, elbow, wrist and finger–the trajectories were averaged and applied to their respective joints similarly for all trials. Each trial was then simulated using the actual, individual trajectory of the fourth variable. Using forward dynamics, the resulting tip speed at contact (when the tip crossed the height of the target) was calculated. An example of a single trial simulation is shown in the animation in the supplemental material (S1 Fig). Regression was used to evaluate the relationship between the fourth variable and tip velocity. This procedure was then repeated to isolate each of the four limb variables. The effects of hand distance and peak height were not considered.

## Results

### Kinematics of the fencing flick

**Fig 4:**
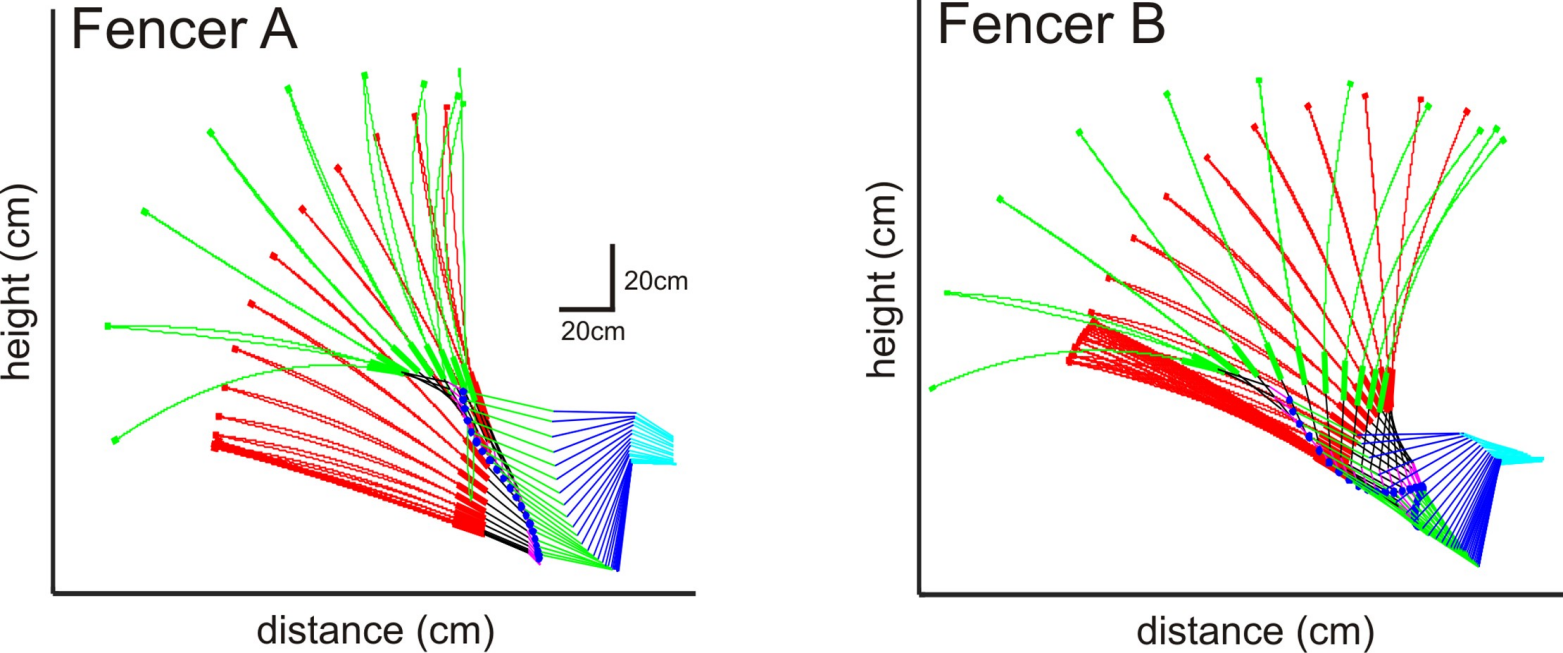
**Stick figure progression of a single, typical flick for both A and B fencers.** The nine tracked features are shown for 35 equally spaced (every 10^th^ frame, or 15 ms) frames. Markers, shown as small filled circles, are connected by linear interpolation for all but the blade between the tip and base (between markers 2 and 3), which is connected by second order polynomial interpolation. The hand, represented by the wrist marker (marker 6), is a larger blue filled circle. Limb segments are identified by color (torso → shoulder, cyan; shoulder → elbow, blue; elbow → writs, green; wrist → hand, magenta; hand to lower blade, back). The blade is colored red for the en garde and backward rotation phase and green for the forward rotation phase of the flick. The tip and blade segments are thickened.

The kinematics of the fencing flick is depicted by video (S2 and S3 Figs) and stick figures (Fig 4) over the course of a representative fencing flick for both fencers A and B. Following the en garde phase, the hand (blue filled circle) moves progressively forward and upward throughout the flick. In parallel, the blade is rotated backward (green blade) by backward joint rotations at shoulder, elbow, wrist and fingers. After reaching a posture at which the tip of the blade reaches nearly vertical or even behind the fencer, the blade is rapidly rotated forward (red blade) by rotation around the finger, wrist and elbow joints, but not the shoulder joint. As the blade approaches the opposing fencer, the forward rotation of all limb joints is rapidly slowed, resulting in the deceleration and curving of the blade toward the target, typically the shoulder or even the back of the opposing fencer.

### Differences between fencers

**Fig 5:**
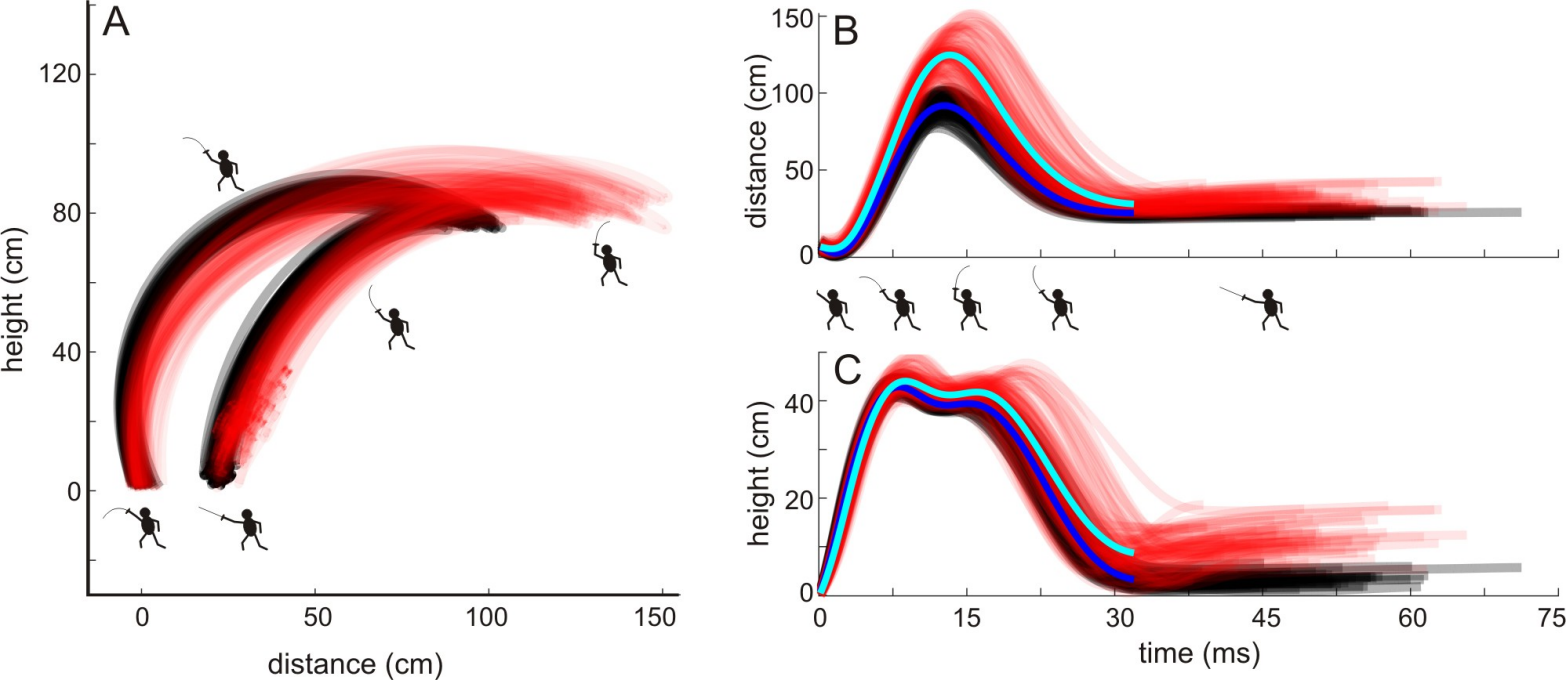
Differences between fencers: Path and trajectories of the foil tip. (A) Paths in the Cartesian coordinate system of the foil tip for all of fencer A’s (n = 67, black) and fencer B’s (n = 78, red) trials. Fencer icons represent the approximate phase of the flick. (B,C) Trajectories of the foil tip over time for both distance (B) and height (C) for both fencers (fencer A black, fencer B red). Time is reversed, progressing from right to left, with the last point on the left immediately before the foil tip contacted with the target. The thick lines represent average trajectory (Fencer A dark blue, Fencer B light blue). The average trajectories are truncated to the duration of the briefest trajectory. Icons indicate approximate progression of the flick.

The primary aim of the flick is to position the tip of the foil on the target with adequate speed to score. Fig 5A shows the path of the tip of the foil for all trials of both fencer A (black) and fencer B (red). Although the initial and final positions of the tip are similar, fencer B moves the tip further back prior to initiating the forward rotation phase of the flick and during the initial forward rotation keeps the tip higher than fencer A. Toward the end of the flick, fencer A moves the tip along an arc higher and more forward than fencer B. The two trajectories, which incorporate time, also differ. Fig 5B shows again that fencer B (red) moves the tip further back during the backward rotation phase and further upward during the forward rotation phase and reaches a peak in height and distance (Fig 5C) earlier than fencer A. Taken together, the fencers differed in both the path and timing of the their foil tips.

**Fig 6:**
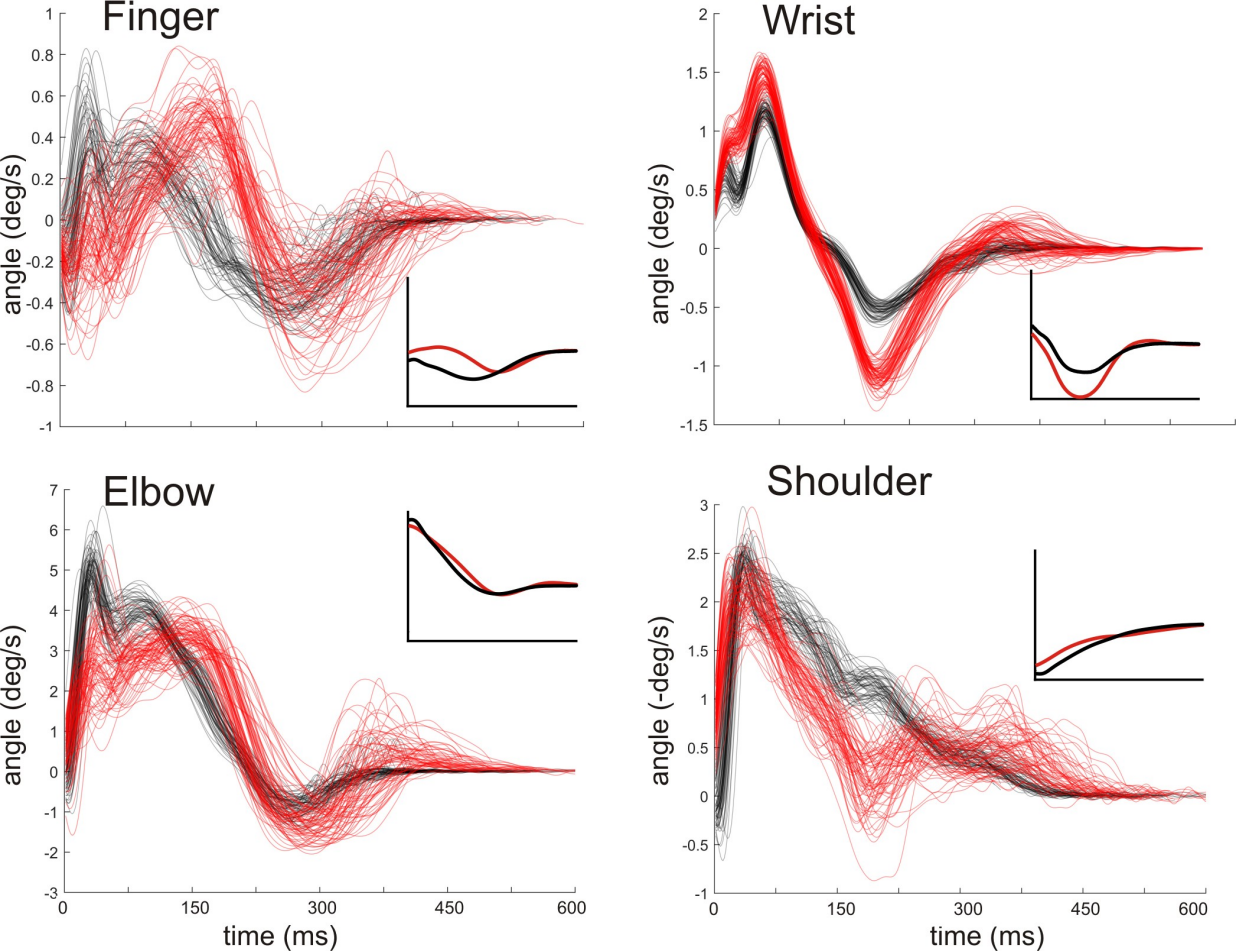
Differences between fencers: Limb joint angular velocity trajectories. Trajectories of finger, wrist, elbow and shoulder joint angle velocities for all of fencer A’s (n = 67, black) and fencer B’s (n = 78, red) trials. Time is reversed, progressing from right to left, with the last point on the left immediately before the foil tip contacted with the target. Shoulder angular velocity axis is reversed. Inset graphs depict mean joint angle for both fencers over time.

**Fig 7:**
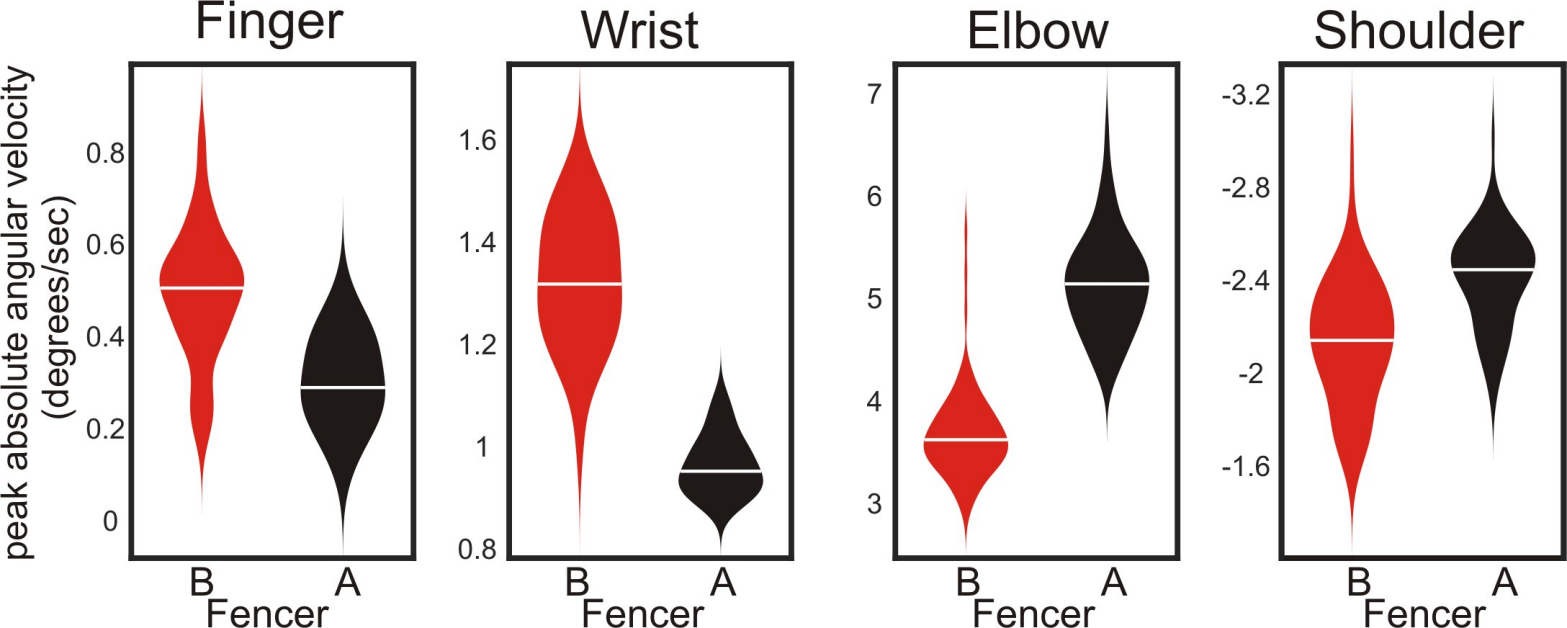
Differences between fencers: Peak limb angular velocity variables. Violin plots show medians and distributions of peak angular velocities around the finger, wrist, elbow and shoulder joints for both fencers A (black) and B (red). Peak velocities were calculated over the full duration except finger peak joint velocity was calculated over frames beyond 60ms (see Fig 5). P-values for all for joints are p<0.000001 (n = 67 for fencer A and n = 78 for fencer B; Mann-Whitney). Effect sizes, expressed as percent differences between medians, are 176%, 138%, 142% and 114% for the finger, wrist, elbow and shoulder joints respectively.

The movement of the blade and tip arises primarily from the angular velocities of the four limb joints–finger, wrist, elbow and shoulder. Fig 6 shows that the four angular velocities for fencer A (black) and fencer B (red) differ in both timing, magnitude and direction. Peak angular velocities of the finger and elbow occur earlier (more to the right) for fencer B than fencer A. Peak angular velocities are greater for fencer B at the wrist. Finally, changes in angular velocity of the shoulder deviate in different directions, with fencer A showing a small increase and fencer B a small decrease in absolute angular velocity. Further, the variability of fencer B’s variables appeared greater than fencer A (e.g. wrist angular velocities). The violin plots in Fig 7 show that the peak angular velocities of fencers A and B, previously shown to differ qualitatively in Fig 6, were also significantly different (p<0.00001 for all for comparisons, Mann-Whitney).

**Fig 8:**
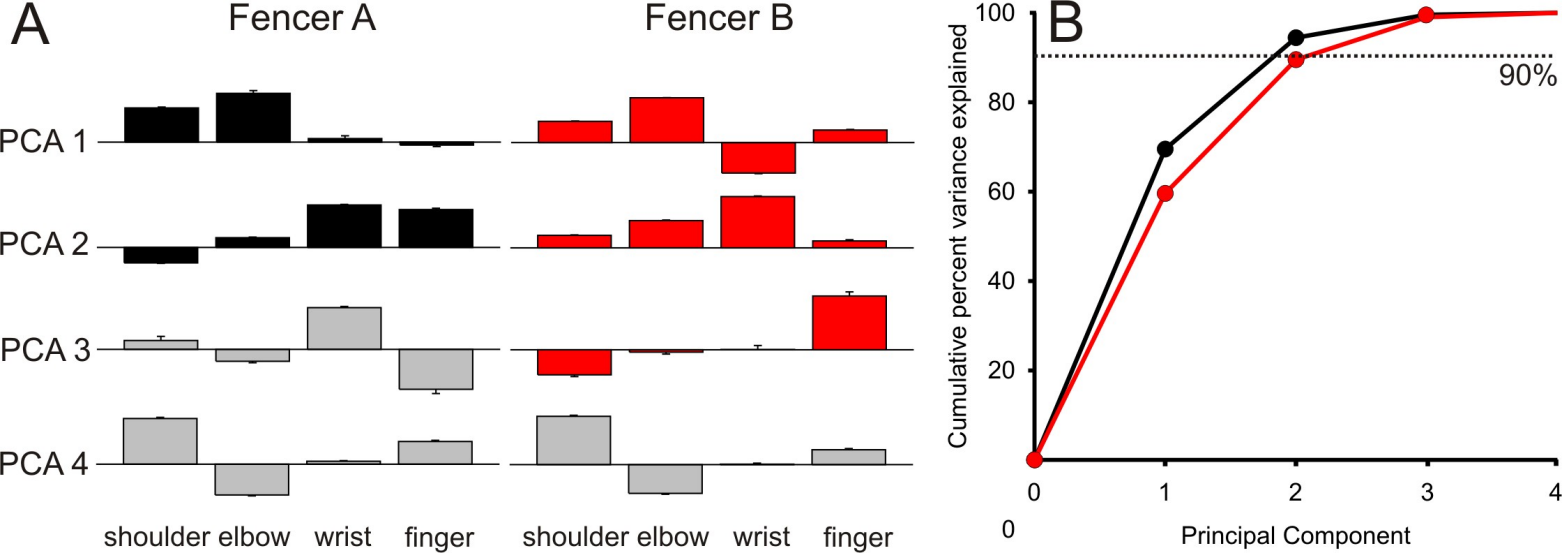
Differences between fencers: Principal component analysis of limb joint angle trajectories. (A) Principal component coefficients (loadings; PCA 1, PCA 2, PCA 3, PCA 4) for the trajectories of the four limb joint angles (finger, wrist, elbow and shoulder) for both fencer A (n = 67 trials) and fencer B (n = 98 trials). Colored (black, red) bars show components retained based on the amount of variance explained (see (B)). Maximum normalized coefficient is 0.86 for the elbow of fencer B. Error bars, which are small, are standard error of the mean. (B) Scree plot shows that the total variance explained by the first two principal components (PCA 1 and PCA 2) for Fencer A (black filled circles) is 94% but for Fencer B (red filled circles) was 89% for two components and 98% for three components. 90% was the cut-off for retaining principal components in (A).

Not only did individual joint angular velocities differ between fencers, but the coordination between joint angular velocities also differed. The results of principal component analysis, which identifies patterns of joint angle changes, is shown in Fig 8. For fencer A, the first two principal components (black) account for 94% of the variance, while for fencer B three principal components (red) were required to reach 90%. The remaining three principal components (gray) were disregarded. There was a striking difference in the patterns of joint contributions to the first two principal components. For fencer A, shoulder and elbow angular velocities dominated the first component while wrist and finger angular velocities dominated the second principal component, suggesting a simple, efficient kinematic strategy. In contrast, for fencer B nearly all four joints contributed to both of the first two principal components, and to a lesser extent the third principal component, suggesting a more complex coordination strategy.

Ultimately, the most important outcome is scoring success. Although similar in ability, fencer A scored significantly more frequently (58%) than fencer A (45%; p = 0.003, Chi^2^, n = 211 for fencer A, 172 for fencer B, including both trials used for analysis and those that were discarded to inforce alternating scoring hits and misses).

### Approach one: Indirect regression

The first approach used to determine the limb variables that influenced scoring success was indirect, in that sequentially the tip variables (distance, velocity) that significantly affect scoring were identified, then the blade variables (peak angular rotation, distance, height) that affect the significant tip variables were identified, and finally the limb variables (distance, height; finger, wrist, elbow, shoulder rotation) that affect the significant blade variables were identified. With this approach, not only were the limb variables that influence scoring revealed, but the intervening tip and blade mechanisms were also identified.

**Fig 9:**
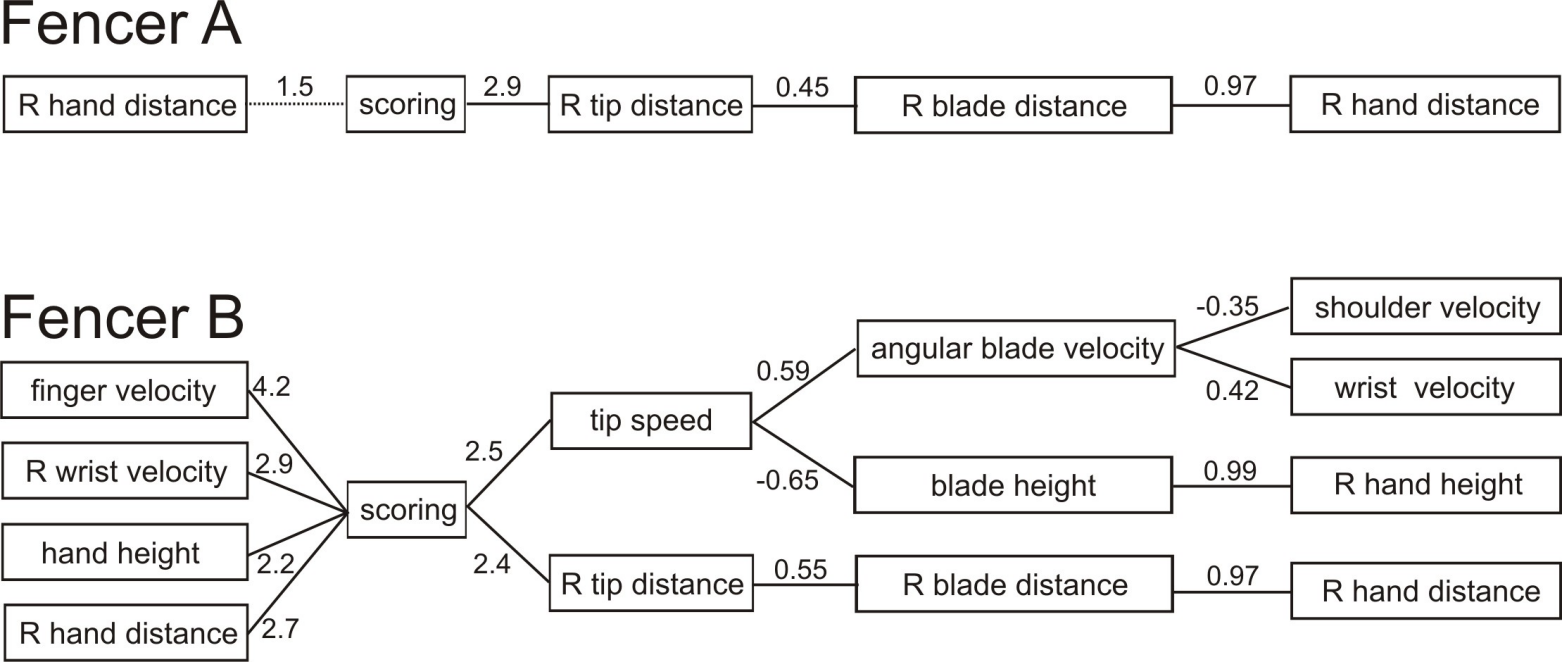
Significant direct and indirect relationships. Diagram depicts significant relationships for both direct (left of “scoring”; limb effects on scoring) and indirect (right of “scoring”; tip effects on scoring, blade effects on tip variables, limb effects on blade variables). Number represent standardized slopes (linear regression) and odds ratios (logistic regression; values less than one are inverted).

There are two ways that any variable, for example final tip distance at contact with the target, could influence another variable, such as scoring. First, increases in tip distance could result in increases in scoring. Regression of the original variables (tip distance versus scoring) would test this hypothesis. Second, deviations from the final tip distance in either direction (closer or further from the target) could result in decreased scoring. To test this possibility, the final tip distance was reflected around the mean distance of successful trials prior to regression. Thus, all variables were tested in both their original and reflected state. The significant dependencies and the magnitude of their effects for approach one are summarized in Fig 9 (to the right of “scoring”).

**Fig 10:**
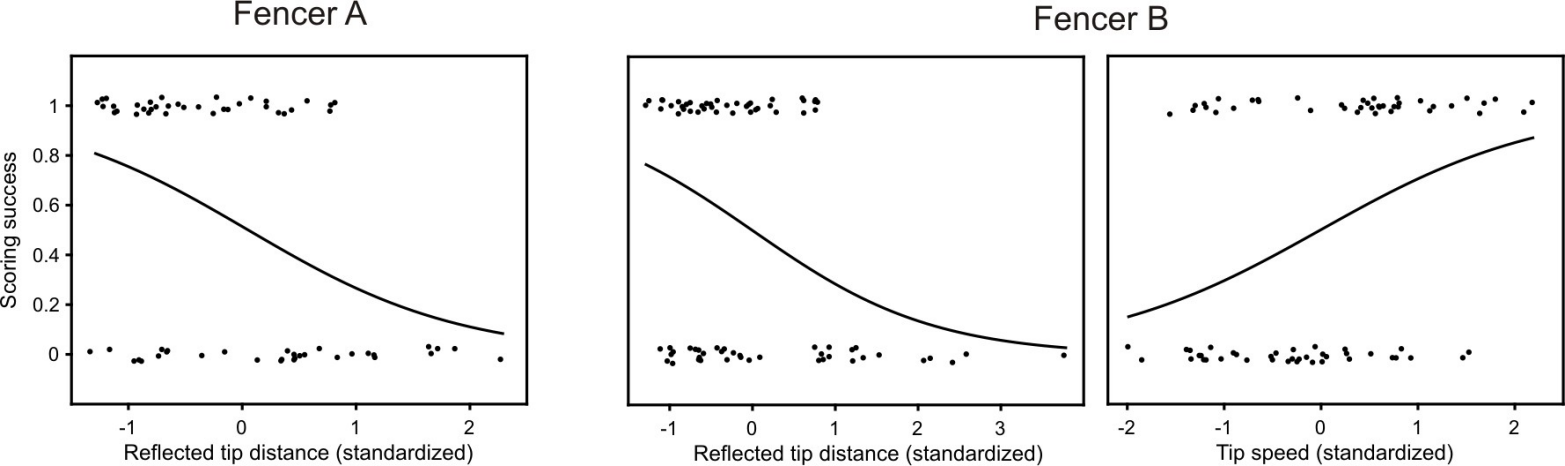
Effects of tip variables on scoring. Dichotomous scoring is jittered and corresponds to either success (1) or a miss (0). Variables are expressed in dimensionless standardized units. The continuous curve correspond to probability. Fencer A: The probability of scoring by fencer A was increased by decreased reflected tip distance (logistic regression, n = 67, r^2^ = 0.24, %explained = 61/77; p = 0.002, odds_std_ = 2.9, r^2^ = 0.24). Not significantly related to scoring (not shown) were tip velocity (p = 0.4), reflected tip velocity (p = 0.1), distance (p = 0.8), tip angle (p = 0.2), and reflected tip angle (p = 1.0). Fencer B: The probability of scoring by fencer B was affected by both reflected tip distance and tip velocity (logistic regression, n = 78, %explained 53/78, r^2^ = 0.29). Decreased reflected tip distance (p = 0.007, odds_std_ = 0.4) and increased tip velocity (p = 0.002, odds_std_ = 2.4) increased scoring probability. Graphs are not partial plots. Not significantly related to scoring were reflected tip velocity (p = 0.4), distance (p = 0.3), tip angle (p = 0.4), and reflected tip angle (p = 0.3).

**Fig 11:**
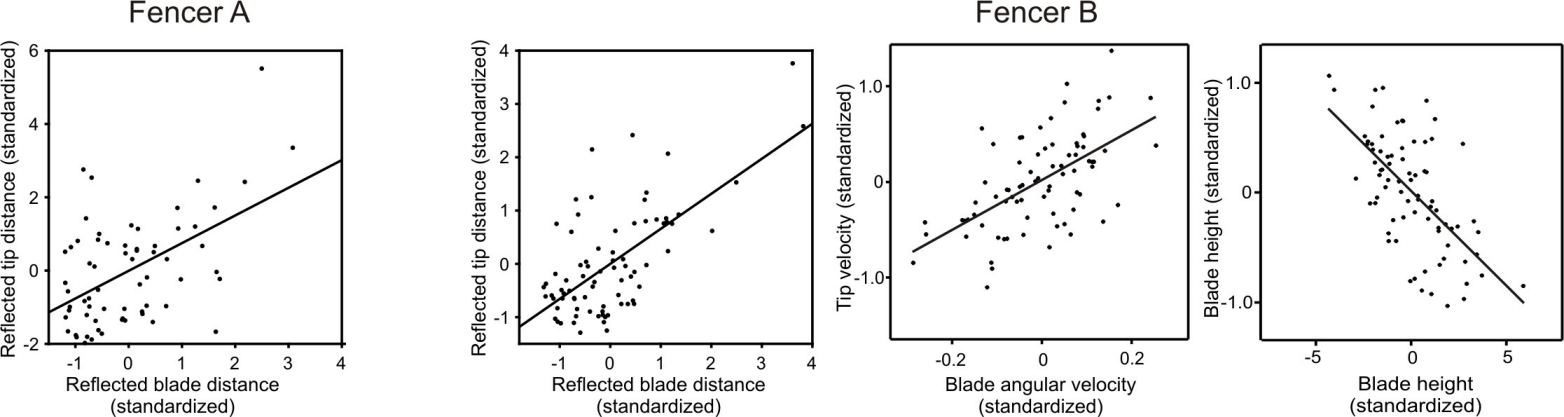
Effects of blade variables on tip variables. Variables are expressed in dimensionless standardized units. Fencer A: Reflected tip distance increased with increased reflected blade distance (linear regression, both variables square root transformed for regression but not plotting, n = 67, p = 0.00016, r^2^ = 0.20, slope_std_ = 0.45, plot). Not significantly related to reflected tip distance (forward step-wise linear regression) were angular blade velocity (p = 0.8), reflected blade velocity (p = 0.2), blade height (p = 0.9), reflected blade height (p = 0.05), and blade distance (p = 0.03). Fencer B. Reflected tip distance increased with increased reflected blade distance (linear regression, both variables square-root transformed for regression, n = 78, p<0.000001, r^2^ = 0.29, slope = 0.62 based on non-transformed data). Not significantly related to blade velocity (p = 0.7), reflected blade velocity (p = 0.6), blade height (p = 0.3), reflected blade height (p = 0.2), or blade distance (p = 0.0005). Tip velocity was affected by both angular blade velocity and blade height (linear regression, n = 78, r^2^ = 0.44). Tip velocity increased with increased angular blade velocity (multiple linear regression, n = 78, p<0.00001, slope_std_ = 0.59, partial plot). Tip velocity increased with decreased blade height (multiple linear regression, n = 78, p<0.00001, slope_std_ = -0.65, partial plot). Not significantly related to tip velocity (forward step-wise linear regression) were reflected angular velocity (p = 0.02), reflected blade height (p = 0.8), blade distance (p = 0.1), reflected blade distance (p = 0.6).

**Fig 12:**
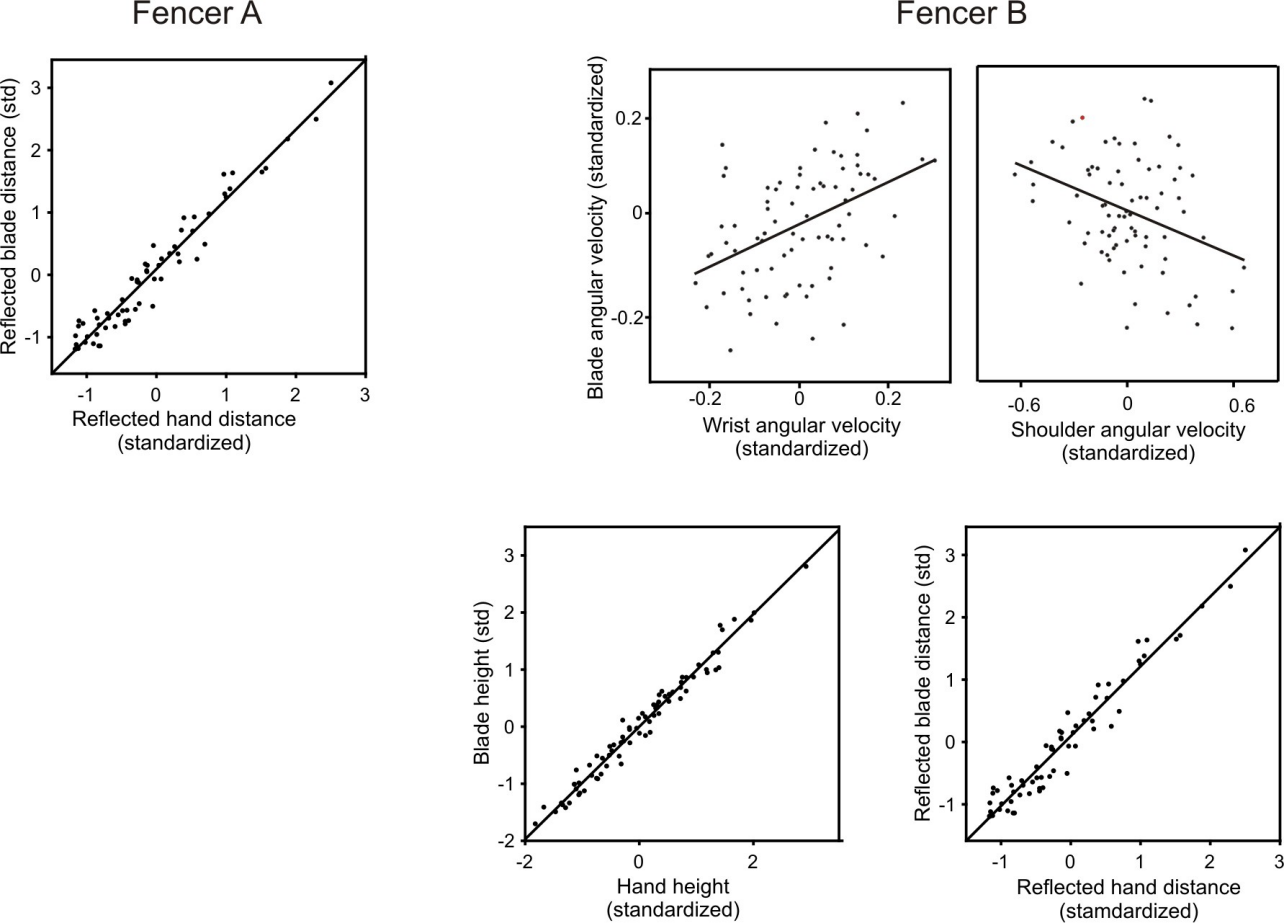
Effects of limb variables on blade variables. Variables are expressed in dimensionless standardized units. Fencer A: Reflected blade distance increased with increased reflected hand distance (linear regression, both variables square root transformed for regression, n = 67, p<0.000001, r^2^ = 0.94, slope_std_ = 0.97). Not significantly related to reflected tip distance (forward step-wise linear regression) were finger velocity (p = 0.5), reflected finger velocity (p = 0.4), wrist velocity (p = 0.6), reflected wrist velocity 0.8), elbow velocity (p = 0.92, reflected elbow velocity (p = 0.8), shoulder velocity (p = 0.6), reflected shoulder velocity (p = 0.3), hand height, (p = 0.9) reflected hand height (p = 0.9), hand distance (p = 0.01). Fencer B: Blade velocity was affected by both wrist and shoulder velocity (linear regression, n = 78, r^2^ = 0.23). Blade velocity increased with increased wrist velocity (linear regression, n = 78, p<0.0002, r^2^ = 0.21, slope_std_ = 0.42, partial plot). Blade velocity increased with increased shoulder velocity (linear regression, n = 78, p<0.001, r^2^ = 0.13, slope_std_ = 0.36, partial plot). Not significantly related to reflected blade velocity (forward step-wise linear regression) were finger velocity (p = 0.11), reflected finger velocity (p = 0.44), reflected wrist velocity (p = 0.99), elbow velocity (p = 0.6), reflected shoulder velocity (p = 0.9), hand height (p = 0.2), reflected hand height (p = 0.4), hand distance (p = 0.2), reflected hand distance (p = 0.32). Blade height increased with increased hand height (linear regression, n = 78, p<0.00001, r^2^ = 0.97, standardized slope = 0.99, plot). Not significantly related to reflected tip distance (forward step-wise linear regression) were finger velocity (p = 0.9), reflected finger velocity (p = 0.3), wrist velocity (p = 0.15), reflected wrist velocity (p = 0.3), elbow velocity (p = 0.35), reflected elbow velocity (p = 0.1), reflected shoulder velocity (p = 0.6), reflected hand height (p = 0.6), hand distance (p = 0.18), reflected hand distance (p = 0.3). Reflected blade distance increased with increased reflected hand distance (linear regression, both variables square root transformed for regression, n = 78, p<0.000001, r^2^ = 0.94, slope_std_ = 0.97 for non-transformed variables, partial plot). Not significantly related to reflected tip distance (forward step-wise linear regression) were finger velocity (p = 0.5), reflected finger velocity (p = 0.8), wrist velocity (p = 0.2), reflected wrist velocity 0.8), elbow velocity (p = 0.2), reflected elbow velocity (p = 0.6), shoulder velocity (p = 0.9), reflected shoulder velocity (p = 0.4), hand height (p = 0.3), reflected hand height (p = 0.1), hand distance (p = 0.003).

Regarding the tip variables that influenced scoring, for fencer A only reflected tip distance at contact significantly affected scoring (p = 0.002, logistic regression, Fig 10). Stepping back to blade variables, again only reflected blade distance affected reflected tip distance (p = 0.0002, linear regression, Fig 11). Finally, only reflected hand distance affected reflected blade distance (p<0.00001, linear regression, Fig 12). Thus, distance but not rotational or height variables, contributed significantly to scoring success.

In contrast, the pattern of significant variables for fencer B that influenced scoring was much more complex. Regarding tip variables that influenced scoring (logistic regression, Fig 10), as with fencer A, reflected tip distance (p = 0.007) influenced scoring (odd ratios 2.4). Stepping back from reflected tip distance, as with fencer A only reflected blade distance affected reflected tip distance (p<0.00001, linear regression, Fig 11) and only reflected hand distance affected blade distance (p<0.00001, linear regression, Fig 12).

Fencer B differed from fencer A in that the tip velocity (p = 0.002) of fencer A also influenced scoring with an effect size (odds ratio 2.5) slightly greater than reflected tip distance (odds ratio 2.4, Fig 10). Steeping back from tip velocity, both angular blade velocity (p<0.00001) and blade height (p<0.00001) influenced tip velocity (linear regression) similarly (slopes 0.59 and -0.65, Fig 11). Interestingly, higher blade height resulted in lower tip velocities. Stepping back to limb variables (linear regression), both wrist (p = 0.002) and absolute shoulder (p = 0.001) angular velocities affected angular blade velocity, while reflected hand height strongly (slope = 0.99) affected blade height (p<0.00001, Fig 12). Peak shoulder velocity, however, was inversely related to tip velocity, presumably because shoulder rotation was strictly backwards throughout the action. Taken together, and in contrast to Fencer A, linear and angular velocities of the tip, blade and limb joints significantly and strongly affected scoring.

### Approach two: Direct regression

**Fig 13:**
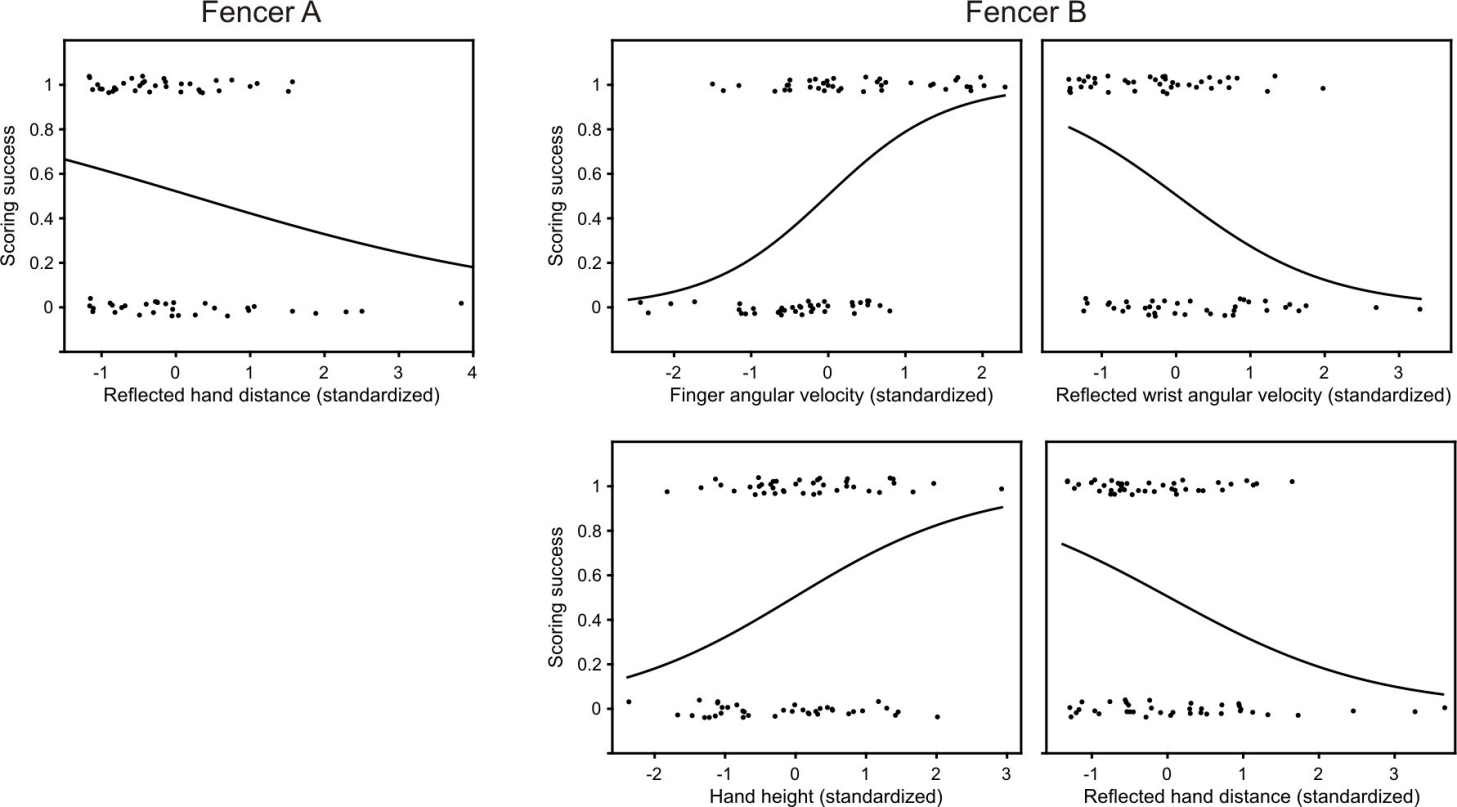
Effects of limb variables on scoring. Dichotomous scoring is jittered and corresponds to either success (1) or a miss (0). Variables are expressed in dimensionless standardized units. The continuous curve correspond to probability. Fencer A: The probability of scoring was increased by decreased reflected hand distance (logistic regression, n = 67, r^2^ = 0.04, %explained = 37/67; p = 0.14, odds_std_ = 0.67). Not significantly related to scoring were finger velocity (p = 0.9), reflected finger velocity (p = 0.4), wrist velocity (p = 0.3), reflected wrist velocity (p = 0.7), elbow velocity (p = 0.4), reflected elbow velocity (p = 0.4), shoulder velocity (p = 0.6), reflected shoulder velocity (p = 0.2), hand height (p = 0.9), reflected hand height (p = 0.3), hand distance (p = 0.3). Fencer B: The probability of scoring was influenced by finger velocity, reflected wrist velocity, hand height and reflected hand distance (logistic regression, n = 78, r^2^ = 0.22, %explained = 8/77). The probability of scoring was increased by finger velocity (logistic regression, n = 78, p = 0.0005, odds_std_ = 3.8). The probability of scoring was decreased by absolute wrist velocity (logistic regression, n = 78, p = 0.003, odds_std_ = 0.37). The probability of scoring was increased by hand height (logistic regression, n = 78, p = 0.009, odds_std_ = 2.2). The probability of scoring was decreased by reflected hand distance (logistic regression, n = 78, p = 0.04, odds_std_ = 0.48). Graphs are not partial plots, thus under-estimating the strength of the relationship. Not significantly related to scoring were reflected finger velocity (p = 0.2), wrist velocity (p = 0.7), elbow velocity (p = 0.3), reflected elbow velocity (p = 0.1), shoulder velocity (p = 0.4), reflected shoulder velocity (p = 0.4), reflected hand height (p = 0.5), and distance (p = 0.3).

The direct approach consisted of using logistic regression to directly identify limb variables that affected scoring. Similar to the indirect approach, in fencer A only reflected hand distance showed even a trend toward explaining scoring (Fig 13; p = 0.14; logistic regression; all other variables were above 0.20). For fencer B, again angular variables of the limb (finger velocity p = 0.0005, odds ratio 4.2; reflected wrist velocity p = 0.003, odds ratio 3.7, hand height, p = 0.009, odds ratio 2.2) as well as reflected hand distance (p = 0.04, odds ratio 2.7) significantly affected scoring, all with broadly similar effect except for hand height, which a weaker effect (Fig 13). The significant dependencies and the magnitude of their effects for approach two are summarized in Fig 9 (to the left of “scoring”).

### Approach three: Computer simulation

In the direct and indirect regression approaches, we used multiple regression to separate out the effects of the six limb variables (shoulder, elbow, wrist and finger angular velocities; hand height and distance). For our third approach, we determined the effects of each of the four angular limb joint variables on tip velocity using a computer simulation to sequentially hold three of the variables constant while allowing the fourth variable to vary. More specifically, mean joint angle trajectories for three variables (e.g. finger, elbow, shoulder angular velocity) and individually varying joint angle trajectories of the fourth variable (e.g. shoulder angular velocity) were applied to a biomechanical model of the foil (see Methods). For each trial, the resulting peak angular velocity of the fourth variable (e.g. shoulder angular velocity) and tip speed at contact were calculated and correlated to determine significant relationships.

**Fig 14:**
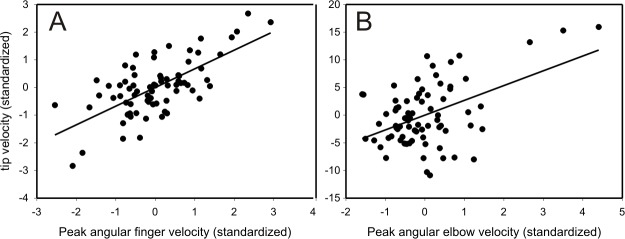
Computer simulation. Relations for significant linear relationships between tip velocity and peak joint velocities. (A) Tip velocity increased significantly with increased finger velocity (p<0.00001, r^2^ = 0.46, slope_std_ = 0.67, linear regression) and (B) increased finger velocity (p = 0.00001, r^2^ = 0.23, slope_std_ = 2.7, linear regression).

As with the direct and indirect regression approaches, fencer A showed no effect of angular limb variables on tip speed. In contrast, and largely consistent with the direct and indirect approaches, fencer B showed a significant relationship between finger (p<0.00001) and elbow (p<0.00001) angular velocities with tip speed (linear regression; Fig 14).

## Discussion

The goal of this research was to identify the kinematic variables, under the fencer’s control, that significantly influence flick scoring success in two elite foil fencers. In particular, we asked what aspect of the movement each *individual* fencer can *change* to improve the likelihood of scoring. Our results showed that the two fencers exhibited significantly different kinematics, coordination and scoring. Using three complementary regression approaches–indirect, direct and computer simulation–we showed that each fencer could improve scoring by changing specific aspects of their kinematics. For fencer A, only improvement in consistency in distance from the target would improve scoring. For fencer B, the changes were more complex. In addition to improvement in consistency in distance, fencer B could also increase finger, wrist and decrease shoulder joint angular velocity or improve consistency of wrist angular velocities. Unexpectedly, and in contrast to common coaching practice, hand height had only a weak effect, possibly because both fencers had learned to keep their hand high at the end of the action. Together, our results emphasize that coaching of elite fencers should be individualized.

### Kinematics of the flick

The flick action begins with a backward rotation of the blade that is produced by backward rotation of all four limb joints–shoulder, elbow, wrist and finger. The angular velocity is primarily determined by the wrist and elbow. After reaching maximum backward rotation, where the blade is vertical or slightly backwards, the elbow, wrist and finger angular, but not shoulder, velocities change direction, propelling the blade forward. The elbow and wrist angular velocities again provide the primary impetus for forward rotation. At end of the action, there is a rapid decrease in the angular velocities of all four joints, causing the blade to bend down and even slightly backward toward the target. The hand moves progressively forward and upwards throughout the action. Although a broadly similar pattern was seen in both fencers, there were differences as shown in Fig 6.

### Flick kinematic differences between fencers

Individual coaching reflects the skills, strength and weakness of the individual fencer; however, only one published study documents differences in kinematics between elite fencers [25]. Although we studied two fencers with similar ability, age and experience, the kinematics, coordination and scoring success of their flicks differed dramatically.

The kinematics of the tip and limb joint angular velocities differed significantly in three ways. First, the path and trajectories of the tip differed between the fencers (Fig 5). Second, fencer B showed consistently and significantly greater peak angular velocities around all four limb joints (Figs 6 and 7). Third, fencer A showed less variance in limb joint angular velocities than fencer B (Figs 6 and 7). Similarly, regression of reflected limb angular velocities revealed that fencer B, but not fencer A, could improve scoring by tightening the wrist angular velocity around the mean. The fencers also differed significantly (58% vs 45% success) in scoring success. Taken together, one explanation for these differences is that the fencers were taught difference patterns of movement and fencer B was not yet as consistent.

The coordination of the limb joints together also differed. Principal component analysis demonstrated that 90% of the variance in fencer A could be explained by two principal components, but fencer B required three principal components. Even more striking, the joint loadings for each principal component different significantly. Fencer A showed a commonly observed shoulder-elbow synergy [35], with little contribution of the wrists and fingers and the 2^nd^ principal component was determined primarily by the wrist and fingers. In contrast, the loadings of the first two principal components for fencer B included contributions of three to four limb joints.

### Individual determinants of scoring success

Our results suggest how each fencer could alter their kinematics to improve scoring success. Fencer A would require few changes to improving scoring success. Across all three approaches, only reflected hand distance was related (significantly for indirect approached, trending for the direct approach) to scoring success (Fig 9, top panel). None of angular limb joint velocities in any of the three approaches were significantly related. Thus coaching advice to fencer A should focus on improving their consistency of distance to the target.

Fencer B was more complex; multiple changes in limb kinematics could improve scoring success (Fig 9, bottom panel). First, as with fencer A, reflected hand distance was highly and significantly correlated with scoring success across both direct and indirect approaches, suggesting the fencer should focus on improving their consistency of distance to the target. Second, for fencer B limb joint angular velocities were positively correlated with scoring for both direct (finger), indirect (shoulder, wrist) and computer simulation (elbow, finger) approaches, and negatively correlated for shoulder (indirect) angular velocity. Although the three approaches yielded somewhat different results, all three identified significant joint angular velocity variables. Thus, fencer B would improve scoring success by increasing finger, elbow and shoulder angular velocities. Since fencer B displayed on average faster joint angular velocities, improvements in scoring based or a further increases in angular velocity is surprising, perhaps reinforcing the dramatically different patterns of coordination used by the two fencers (described above). Second, reflected wrist velocity was correlated with scoring in the direct approach, suggesting that greater consistency in wrist velocity would improve scoring. This result is consistent with overall greater variability displayed by fencer B. Third, hand height was also correlated directly with scoring success, although the magnitude of the effect (odds_std_ = 2.2) was the smallest of the four correlated variables (odd_std_ varied from 2.7 to 4.2 for the other three variables).

One unexpected result was the general lack of or weak effect of hand height, which is strongly emphasized in coaching practice and constituted our primary hypothesis. One explanation arises from the experimental design coupled to the likelihood that both fencers, being experienced with the flick and having extensive coaching on hand height, were largely keeping their hand height high enough at the end of the action. In other words, our regression analysis answers the question as to what the fencers can *change* to improve scoring success. If hand height is already *consistently* adequate, then higher hand height would not be beneficial.

### Implications for fencing research

Beyond our primary aim of providing the first description of the kinematics of flick, our experimental approach is novel in two ways. First, we distinguished between the *linear effects* and *consistency* of each variable by including both the variable and its reflection around the mean (of successful trials) in the regression analysis. For example, significant correlations were obtained for angular finger velocity (linear effect) and reflected angular wrist velocity (consistency). We found no similar approach in the literature.

Second, while computer simulations are commonly incorporated into kinematic and kinetic analysis of sports movement, there is only one simulation study for fencing. Further, we believe that we have adopted a unique approach that separates the effects individual variables. While multiple regression can accomplish the same effect, non-linear or interaction relations complicate interpretation. In contrast, we believe our approach may be less sensitive to nonlinearities, and in any event provide an alternative, complementary approach to identifying significant variables.

An unexpected finding was the clear differences between fencers in their patterns of joint coordination revealed by principal component analysis. Although employed in the study of other sports to identify synergies, ours is the first to identify joint synergies underlying a fencing action. Although studies of limb kinematic and tool usage synergies have become common in the motor control literature, we have failed to find literature that addresses synergies for the use of *flexible sports tools*, such as the fencing blade. Further research could provide additional insight in to the normal coordination of human movement.

### Implications for coaching of fencers

The second implication of our research was to inform coaching and teaching methods of the flick throughout the fencing community. Our results showed that hand height was not as important as other variables, falsifying our original hypothesis. However, because our study treated each fencer individually, other fencers may benefit from increased hand height. The broader implication is that in group lessons of elite fencers, emphasis on movements such as hand might not be appropriate because *they have already mastered that skill*. Rather, emphasis should be placed on movements, such as distance to the target that may more uniformly influence success across fencers. Regarding individual coaching, which is more important to elite fencers, instruction should be matched to abilities, consistency and kinematics of each individual fencer. While not a new concept, the dramatic difference between the studied fencers in their execution of the flick emphasizes the need for individualization in coaching.

In order to closely control relevant variables, our experiments were conducted with foil fencing and in a constrained environment using a dummy opponent, simplified target and absence of other aspects of a conventional bout. Further, only two, high level, participants were studied in two rather than three dimensions. Consequently, extrapolation of our results to fencing coaching should be done cautiously. In the future, studies of conventional bouts [18,36,37] might clarify the practical implications of our results.

Fencing is ideal for the real-time use of motion capture to inform coaching because the actions are largely planar and restricted in space. Although our analyses were completed off-line, all three approaches could potentially be executed in real-time to provide immediate feedback to the fencer and coach.

## Supporting information

S1 Fig Animation of computer simulated flick. Shoulder, elbow, and wrist angular rotations were held constant at their mean trajectories. Finger angular velocity was varied; animation illustrates one of the 67 trials for fencer B (11 frames, mp4, 60 KB).
(MP4)

S2 Fig Video of Fencer A. Typical trial showing flick in slow motion (650 fps, mp4, 8.7 MB, 375 frames).
(MP4)

S3 Fig Video of Fencer B. Typical trial showing flick in slow motion (650 fps, mp4, 6.5 MB, 365 frames).
(MP4)

S1 Data Computed variables used in regression. (XLSX)

## References

[1] Roi GS, Bianchedi D. The science of fencing. Sports Med. 2008;38(6):465–481. doi: 10.2165/00007256-200838060-00003 18489194

[2] Handelman R. Fencing: A Practical Guide for Training Young Athletes: Pattinando Publishing; 2010.

[3] Murgu A. Fencing. Phys Med Rehabil Clin N Am. 2006;17(3):725–736. 10.1016/j.pmr.2006.05.008 16952760

[4] Green W. Distinguishing Characteristics of Classical Fencing when Compared to Modern Fencing. Classical Academy of Arms. 2016.

[5] Chen TL, Wong DW, Wang Y, Ren S, Yan F, Zhang M. Biomechanics of fencing sport: A scoping review. PloS ONE. 2017;12(2): e0171578 10.1371/journal.pone.0171578 28187164 PMC5302478

[6] Harmer PA. Getting to the point: injury patterns and medical care in competitive fencing. Current sports medicine reports. 2008;7(5):303–307. 10.1249/JSR.0b013e318187083b 18772692

[7] Sinclair J, Bottoms L, Taylor K, Greenhalgh A. Tibial shock measured during the fencing lunge: the influence of footwear. Sports Biomech. 2010;9(2):65–71. 10.1080/14763141.2010.491161 20806842

[8] Greenhalgh A, Bottoms L, Sinclair J. Influence of surface on impact shock experienced during a fencing lunge. J Appl Biomech. 2013;29(4):463–467. 22923353 10.1123/jab.29.4.463

[9] Turner A, James N, Dimitriou L, Greenhalgh A, Moody J, Fulcher D, et al Determinants of Olympic fencing performance and implications for strength and conditioning training. J Strength Cond Res. 2014;28(10):3001–3011. 10.1519/JSC.0000000000000478 24714533

[10] Sinclair J, Bottoms L. Gender specific ACL loading patterns during the fencing lunge: Implications for ACL injury risk. Science & Sports. 2018 10.1016/j.scispo.2018.05.005

[11] Bauchmoyer SL, Lefevers V. Relationships Between Components of Speed, Accuracy, and Fencing Success. International Symp on Biomech Sports. 1975:1–8.

[12] Stewart Kerry J, Peredo Alfred R, Williams CM. Physiological and morphological factors associated with successful fencing performance. J Hum Ergol. 1977;6(1):53–60. 10.11183/jhe1972.6.53 608957

[13] Sapega AA, Minkoff J, Nicholas JA, Valsamis M. Sport-specific performance factor profiling: Fencing as a prototype. Am J Sports Med. 1978;6(5):232–235. 10.1177/036354657800600504 707682

[14] Cronin J, McNair P, Marshall R. Lunge performance and its determinants. J Sports Sci. 2003;21(1):49–57. 10.1080/0264041031000070958 12587891

[15] Sinclair JK, Bottoms L. Gender differences in the Achilles tendon load during the fencing lunge. Balt J Health Phys Activity. 2014;6(3):199 10.2478/bjha-2014-0018

[16] Sinclair J, Bottoms L. Gender differences in patellofemoral load during the epee fencing lunge. Res Sports Med. 2015;23(1):51–58. 10.1080/15438627.2014.975813 25630246

[17] Bottoms L, Greenhalgh A, Sinclair J. Kinematic determinants of weapon velocity during the fencing lunge in experienced épée fencers. Acta Bioeng Biomech. 2013;15(4):113 10.5277/abb130414 24479483

[18] Zhang B, Chu D, Hong Y. Biomechanical analysis of the lunge technique in the elite female fencers. International Symp on Biomech Sports. 1999;1(1):65–68.

[19] Gholipour M, Tabrizi A, Farahmand F. Kinematics analysis of lunge fencing using stereophotogrametry. World J Sport Sci. 2008;1(1):32–37.

[20] Guan Y, Guo L, Wu N, Zhang L, Warburton DER. Biomechanical insights into the determinants of speed in the fencing lunge. Eur J Sports Sci. 2018;18(2):201–208. 10.1080/17461391.2017.1414886 29249174

[21] Singer RN. Speed and Accuracy of Movement as Related to Fencing Success. Res Q Am Assoc Health Physical Ed. 1968;39(4):1080–1083. 10.1080/10671188.1968.10613465 5250084

[22] Harmenberg J, Ceci R, Barvestad P, Hjerpe K, Nyström J. Comparison of different tests of fencing performance. Age. 1991;23:21–30. 10.1055/s-2007-1024736 1797700

[23] Williams L, Walmsley A. Response timing and muscular coordination in fencing: a comparison of elite and novice fencers. J Sci Med Sports. 2000;3(4):460–475. 10.1016/S1440-2440(00)80011-0 11235010

[24] Klinger A, Adrian M, Dee L. Effect of Pre-Lunge Conditions on Performance of Elite Female Fencers. 1985.

[25] Frère J, Göpfert B, Nüesch C, Huber C, Fischer M, Wirz D, et al Kinematical and EMG-classifications of a fencing attack. Int J Sports Med. 2011;32(01):28–34. 10.1055/s-0030-1267199 21086241

[26] Gresham-Fiegel CN, House PD, Zupan MF. The effect of nonleading foot placement on power and velocity in the fencing lunge. J Strength Cond Res. 2013;27(1):57–63. 10.1519/JSC.0b013e31824e0e9d 22395272

[27] Zhang D, Ding H, Wang X, Qi C, Luo Y. Enhanced response inhibition in experienced fencers. Sci Rep. 2015;5:16282 10.1038/srep16282 26541899 PMC4635338

[28] Gutiérrez-Cruz C, Rojas FJ, Gutiérrez-Davila M. Effect of defence response time during lunge in foil fencing. J Sports Sci. 2016;34(7):651–657. 10.1080/02640414.2015.1068434 26185981

[29] Gutiérrez-Davila M, Rojas FJ, Gutiérrez-Cruz C, Navarro E. Effect of dual-attention task on attack and defensive actions in fencing. European Journal of Sport Science. 2017;17(8):1004–1012. 10.1080/17461391.2017.1332100 28562182

[30] Sinclair J, Bottoms L. Differences in Limb and Joint Stiffness during the Fencing Lunge. Cent Eur J Sport Sci Med. 2015;11(3):39–44. 10.18276/cej.2015.3-04

[31] Garret MR, Kaidanov EG, Pezza GA. Foil, Saber, and Épée Fencing: Skills, Safety, Operations, and Responsibilities State College, PA: Penn State Press; 1994.

[32] Barth B, Beck E. The complete guide to fencing. Aachen, Germany: Meyer & Meyer Verlag; 2007.

[33] Goriely A, McMillen T. Shape of a cracking whip. Phys Rev Lett. 2002;88(24):244–301. 10.1103/PhysRevLett.88.244301 12059302

[34] Robson JM. The physics of fly casting. American Journal of Physics. 1990;58(3):234–240. 10.1119/1.16191

[35] Inouye JM, Valero-Cuevas FJ. Muscle synergies heavily influence the neural control of arm endpoint stiffness and energy consumption. PLoS Comput Biol. 2016;12(2):e1004737 10.1371/journal.pcbi.1004737 26867014 PMC4750997

[36] Aquili A, Tancredi V, Triossi T, De Sanctis D, Padua E, D'arcangelo G, et al Performance analysis in saber. J Strength Cond Res. 2013;27(3):624–630. 10.1519/JSC.0b013e318257803f 23443217

[37] Wylde JM, Tan FHY, O’Donoghue GP. A time-motion analysis of elite women’s foil fencing. Int J Perf Anal Sport&nbsp. 2013;13(2):365–376. 10.1080/24748668.2013.11868654

